# Ultrahigh Strength and Exceptional Work Hardening in a Hierarchical‐Structured Alloy via Hetero‐Interface‐Mediated Twinning

**DOI:** 10.1002/advs.202509584

**Published:** 2025-07-13

**Authors:** Yitong Yang, Jingyu Pang, Hongwei Zhang, Jiajia Shen, Zhenqiang Xing, Yuan Sun, Aimin Wang, J. P. Oliveira, Wei Wang, Zengbao Jiao

**Affiliations:** ^1^ Shi‐changxu Innovation Center for Advanced Materials Institute of Metal Research Chinese Academy of Sciences 72 Wenhua Road Shenyang 110016 China; ^2^ School of Material Science and Engineering University of Science and Technology of China 72 Wenhua Road Shenyang 110016 China; ^3^ CENIMAT/I3N Department of Materials Science NOVA School of Science and Technology Universidade NOVA de Lisboa Caparica 2829‐516 Portugal; ^4^ Department of Mechanical Engineering Research Institute for Advanced Manufacturing The Hong Kong Polytechnic University Hong Kong 999077 China; ^5^ The Hong Kong Polytechnic University Shenzhen Research Institute Shenzhen 518057 China

**Keywords:** heterostructure, high entropy alloy, mechanical property, work hardening

## Abstract

Yield strength and work hardening are two critical mechanical properties of metallic structural materials. However, increasing yield strength through conventional strengthening mechanisms often restricts further dislocation multiplications and interactions, which significantly reduces work hardening and poses a challenge to achieving an optimal balance between these properties in material design. Here, an innovative approach to simultaneously enhance both yield strength and work hardening in a heterostructured, nanoprecipitation‐strengthened alloy is reported. This alloy exhibits an exceptional combination of a yield strength exceeding 1.5 GPa and an ultrahigh work hardening rate of 6 GPa, resulting in an extremely high tensile strength of 2.2 GPa and a uniform ductility of 20%. The ultrahigh yield strength primarily stems from nanoprecipitates and ultrafine grains, while the exceptional work hardening mainly originates from hetero‐interface‐mediated twinning. The hetero‐deformation between the coarse‐grained and ultrafine‐grained regions results in dislocation pile‐ups and strain gradients near the interfaces, which provides the ultrahigh stress necessary to activate mechanical twinning, thereby substantially improving the work hardening and plastic deformation stability of the alloy. The hetero‐interface architecting strategy can potentially be applied to numerous other alloys, paving the way for designing novel materials with unprecedented mechanical properties for technological applications.

## Graphical abstract

This study presents a novel strategy for achieving exceptional work hardening in ultrahigh‐strength alloys by designing hierarchically heterogeneous dual‐phase (HHDP) structures, where hetero‐deformation‐induced strain gradients activate deformation twinning. The resulting alloy exhibits an unprecedented combination of ultrahigh yield strength (> 1.5 GPa) and outstanding work hardening rate (6 GPa), imparting a tensile strength of ≈2.2 GPa and a uniform ductility of ≈20%.

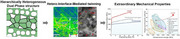

## 1 Introduction

Ultrastrong‐yet‐ductile materials are highly desirable for a wide range of applications, such as aerospace, automotive, construction, and energy industries.^[^ ^1^, ^2^, ^3^ ^]^ However, there is a mutually exclusive relationship between strength and ductility, known as the strength‐ductility trade‐off.^[^ ^1^, ^4^, ^5^, ^6^ ^]^ Generally, introducing crystal defects, such as precipitates and grain boundaries, can impede dislocation motion, thereby increasing the yield strength of materials. Unfortunately, the resulting lack of dislocation mobility and storage capacity can lead to severe strain localization,^[^ ^7^, ^8^ ^]^ causing the material to lose tensile ductility. Essentially, the degradation of tensile ductility in metallic materials at high flow stresses is mainly due to an increased propensity of severely localized deformation, which results in early plastic deformation instability.^[^ ^9^, ^10^ ^]^ According to Hart's criterion, only when the work hardening rate remains sufficiently high is the subsequent tensile flow stable at high yield strengths.^[^ ^11^, ^12^ ^]^ Therefore, enhancing the work hardening capability is crucial for improving the ductility of high‐strength materials, which represents a central issue in the development of high‐performance structural materials.

Considerable effort has been devoted to enhancing the synergy between high yield strength and work hardening capability. Deformation twinning has been demonstrated to improve the work hardening and tensile ductility of metals and alloys with low stacking fault energies (SFEs), a phenomenon known as the twinning‐induced plasticity (TWIP) effect. For example, Yoshida et al.^[^ ^13^ ^]^ inhibited the cross‐slip of dislocations and promoted the formation of deformation twins in a Co_20_Cr_40_Ni_40_ alloy by regulating the SFE, thereby significantly improving the work hardening ability of the material. Through atomic‐resolution in situ experiments, Ma et al.^[^ ^14^ ^]^ revealed that the slip of full dislocations is hindered by twin boundaries in an Fe_48_Mn_32_Co_10_Cr_10_ alloy, which effectively suppresses the dynamic recovery of dislocations and enhances the work hardening of the alloy. Unfortunately, the yield strength of most TWIP materials is only moderate (less than 1000 MPa), because in low SEF materials, dislocations easily dissociate and undergo planar slip, lacking sufficient structural barriers to hinder their motion. To enhance the yield strength of TWIP alloys, researchers have explored incorporating additional strengthening mechanisms, such as precipitation strengthening and grain boundary strengthening, which can elevate the yield strength of alloys to over 2000 MPa. However, the introduction of these crystal defects generally hinders the twinning process. Twinning proceeds via the nucleation, propagation, and growth of irregular, 3D domains that develop through a combination of lattice shear and atomic shuffling. Crystal defects, such as precipitates and grain boundaries, can significantly hinder the propagation of twins and reduce the TWIP effect, thereby decreasing the work hardening capability of the alloys. Therefore, achieving a strong synergy between high yield strength and work hardening capability remains extremely challenging.

In this work, we report an innovative approach to achieving exceptional work hardening capabilities in ultrahigh‐strength alloys by designing hierarchically heterogeneous dual‐phase (HHDP) structures, which incorporate multiscale grain structures and precipitates. The coarse grains have a face‐centered cubic (FCC) structure and contain high number densities of L1_2_ nanoprecipitates, whereas the ultrafine‐grained regions consist of ultrafine FCC and L1_2_ grains. This combination provides strong strengthening effects, leading to an ultrahigh yield strength of more than 1.5 GPa. Intriguingly, hetero‐deformation between the coarse‐grained and ultrafine‐grained regions induces a strain gradient near their interface, which effectively triggers deformation twinning in the interface‐affected zone, thereby leading to exceptional work hardening with a superior work hardening rate (6 GPa). This unique deformation mechanism enables our alloy to achieve a tensile strength of over 2.2 GPa and a uniform ductility of ≈20%, outperforming conventional alloys reported in the literature. Therefore, through the HHDP strategy, we have overcome the trade‐off between yield strength and work hardening capability, leading to the development of a new class of structural materials with ultrahigh yield strength, high uniform ductility, and significant work hardening capacity. This strategy provides a novel solution to the strength‐ductility trade‐off in ultrahigh‐strength materials.

## 2 Results

### 2.1 Microstructural Characterization

**Figure 1:**
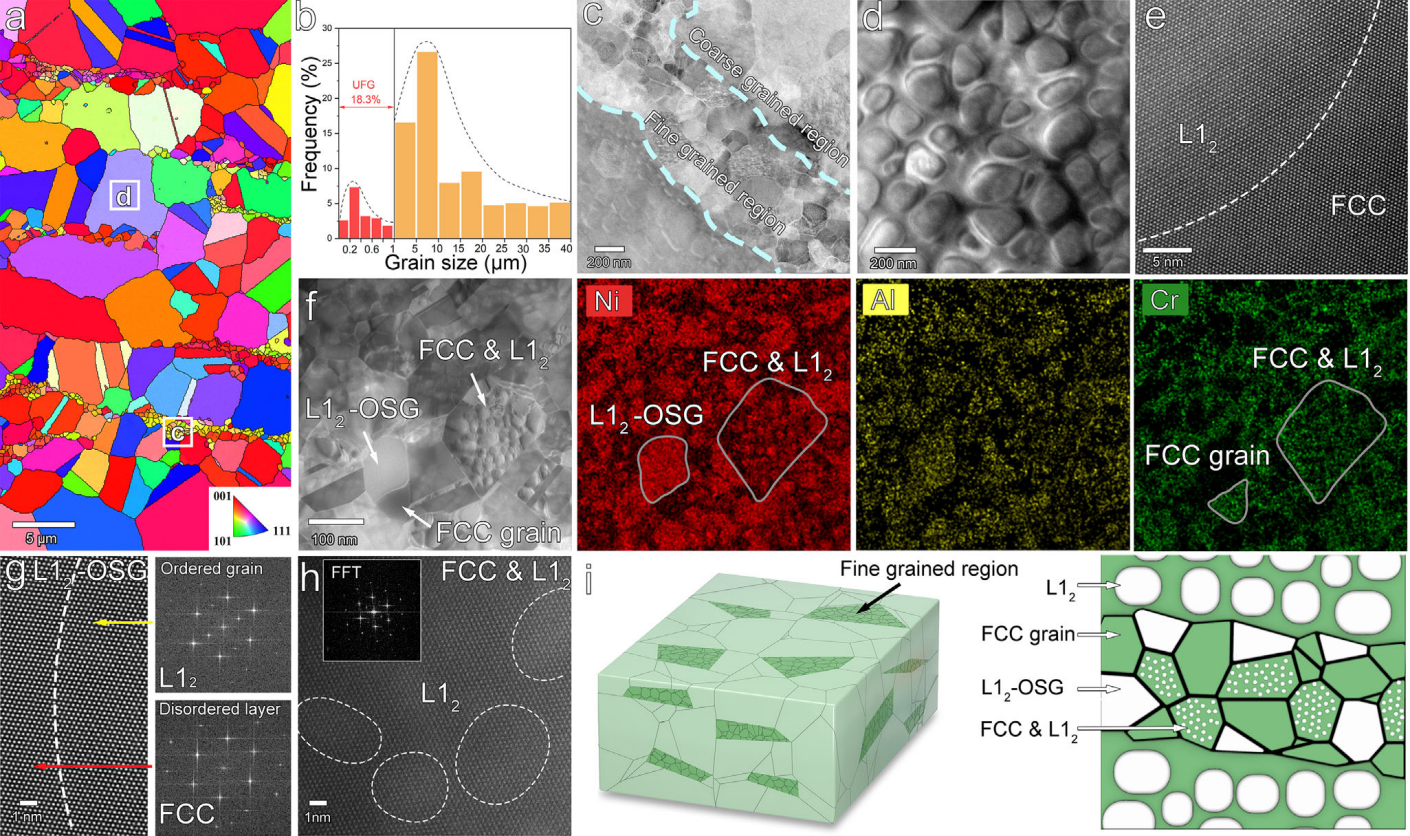
Hierarchically heterogeneous microstructure of the HHDP alloy. a) EBSD inverse pole figure (IPF) map illustrating the heterogeneous grain structure, which includes both coarse‐grained and ultrafine‐grained regions. b) Histogram depicting the grain size distribution. c) HAADF‐STEM image highlighting the coarse‐grained and ultrafine‐grained regions. d,e) HAADF‐STEM and HR‐STEM images of a coarse‐grained region, demonstrating the uniform precipitation of coherent L1_2_ nanoparticles within the FCC grains. f) HAADF‐STEM and EDS maps of an ultrafine‐grained region, showing three types of ultrafine grains: L1_2_‐ordered grains, FCC grains containing L1_2_ nanoparticles, and FCC grains without precipitates. g) HR‐STEM image of an L1_2_‐ordered ultrafine grain, featuring a disordered intergranular nanolayer at grain boundaries. h) HR‐STEM image of an ultrafine FCC grain containing L1_2_ nanoparticles. i) Schematic illustration of the unique microstructure of the HHDP alloy.

Microstructural characterization reveals that our HHDP alloy exhibits hierarchically heterogeneous structures in both grains and precipitates. Electron backscattered diffraction (EBSD) analysis shows that the HHDP alloy has a distinct heterogeneous grain structure, consisting of coarse‐grained and ultrafine‐grained regions, with volume fractions of 82% and 18%, respectively (**Figure** **1**a). The coarse‐grained region exhibits an equiaxed structure with an average grain size of 6.9 ± 2.2 µm and a nearly uniform distribution of crystallographic orientations (Figure S1a), indicating the absence of noticeable texture. In contrast, the ultrafine‐grained regions are distributed in a necklace‐like pattern at the boundaries of the coarse grains, with an average grain size of 242 ± 37 nm (Figure 1b,c) and a slightly preferred orientation along the <001> crystal direction (Figure S1b). Energy‐dispersive spectroscopy (EDS) reveals that the chemical compositions of the coarse‐grained and ultrafine‐grained regions are nearly identical within measurement uncertainties (Table S1), demonstrating the absence of macro‐segregation. Transmission electron microscopy (TEM) indicates that high‐density nanoparticles, with an average size of 68 ± 13 nm, are uniformly distributed within the matrix of the coarse grains (Figure 1d,e; Figure S2). High‐angle annular dark‐field scanning TEM (HAADF‐STEM) and corresponding fast Fourier transform (FFT) patterns illustrate that the nanoparticles have an L1_2_ structure and are coherent with the FCC matrix. EDS reveals that these nanoparticles are enriched in Ni, Co, Al, and Ti, with an atomic ratio of (Ni + Co): (Al + Ti) close to 3, suggesting that these nanoparticles are of the (Ni,Co)_3_(Al,Ti)‐type (Figure S2). The ultrafine grains form a strip‐like zone with a width of ≈700 nm along the boundaries of some coarse grains. Intriguingly, three categories of structures are present in this ultrafine‐grained region (Figure 1f): 1) L1_2_‐ordered superlattice grains (OSGs) enriched in Ni, Co, Al, and Ti; 2) FCC grains enriched in Co, Fe and Cr without any precipitates; and 3) FCC grains containing L1_2_ nanoparticles. Disordered intergranular nanolayers can be observed at the grain boundaries of OSGs (Figure 1g), and the average size of L1_2_ nanoparticles precipitated in FCC grains is ≈4 nm, which maintains good coherence with the matrix (Figure 1h). For a clear demonstration of the multi‐scale heterostructure of the alloy, we show a schematic microstructure diagram in Figure 1i. The ultrafine‐grained regions are distributed in a strip pattern along the boundaries of the coarse‐grained regions. Within the ultrafine‐grained regions, the FCC and L1_2_ phases exhibit three distinct distribution modes: L1₂‐ordered ultrafine grains, FCC ultrafine grains containing L1_2_ nanoparticles, and FCC grains with no precipitates.

### 2.2 Extraordinary Mechanical Performance

**Figure 2:**
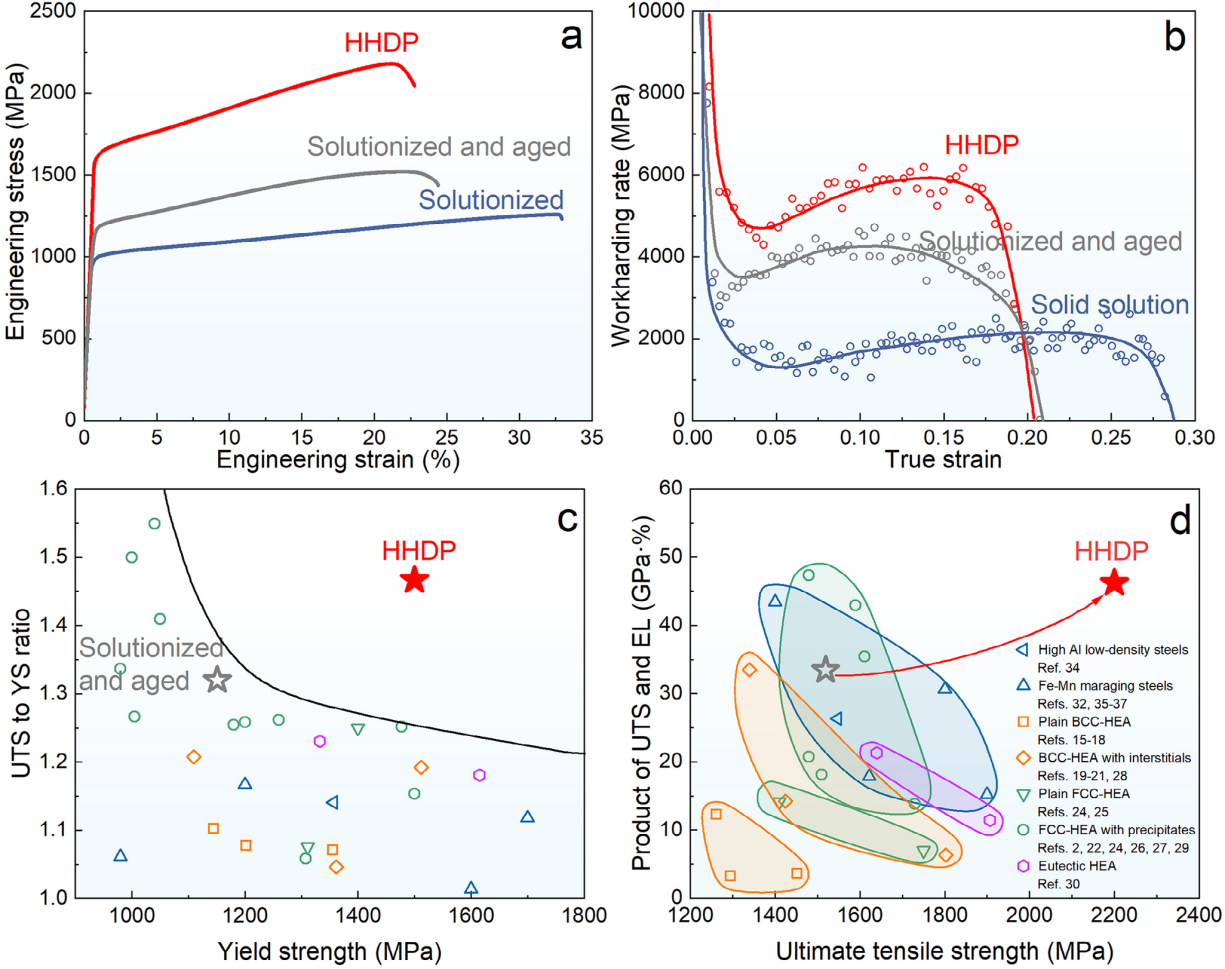
Mechanical performance of the HHDP alloy. a) Tensile stress–strain curves of the HHDP alloy (red) at room temperature, with curves for the as‐solutionized (blue) and conventionally solutionized and aged (gray) samples included for comparison. b) Work hardening rate curves of the HHDP alloy and reference alloys. c) Comparison of the ultimate tensile strength (UTS) to yield strength (YS) ratio versus yield strength for the HHDP alloy and other high‐performance alloys. Mechanical property data of these high‐performance alloys are provided in Table S1. d) Comparison of the product of UTS and uniform elongation (EL) versus ultimate tensile strength for the HHDP alloy and other high‐performance alloys in the literature.

We evaluated the mechanical properties of the HHDP alloy using tensile tests at room temperature. For comparison, two reference materials with the same composition but without hierarchically heterogeneous structures were also tested: an as‐solutionized sample (Figure S3a) and a conventionally solutionized and aged sample (Figure S3b). Representative engineering stress–strain curves of the three samples are shown in **Figure** **2**a. The HHDP alloy exhibits a remarkable yield strength of ≈1.53 ± 0.12 GPa, which is much higher than that of the two reference materials (1.15 GPa for the as‐solutionized sample and 0.96 GPa for the solutionized and aged sample). The slight <001> texture in the ultrafine‐grained regions implies that the alloy may exhibit some degree of mechanical anisotropy. However, given that the ultrafine‐grained regions constitute only 18% of the volume fraction and possess significantly higher strength than the coarse grains, the influence of this texture on the overall anisotropy of yield strength is expected to be limited. After yielding, the stress of the HHDP alloy increases drastically with strain, achieving an ultimate tensile strength of ≈2.2 GPa. The difference between the yield strength and ultimate tensile strength of the HHDP alloy is more than 700 MPa, illustrating pronounced work hardening at an ultrahigh yield strength level. In contrast, the two reference materials show only a slight increase in stress after yielding, indicating weak work hardening capability. Additionally, despite a drastic increase in both yield strength and ultimate tensile strength, the HHDP alloy remains highly ductile, exhibiting a uniform elongation of over 20%. The work hardening rate curves of the HHDP alloy and the two reference materials are displayed in Figure 2b. Intriguingly, the HHDP alloy exhibits multi‐stage work hardening behavior. After an initial drop associated with the elastic‐to‐plastic transition, the work‐hardening rate increases substantially to 6 GPa at the 10% strain and remains stable over the 10–17% strain range. In contrast, the work hardening rate of the conventionally solutionized and aged sample reaches 4 GPa and decreases after the 10% strain, whereas the as‐solutionized sample shows a low work hardening rate of 2 GPa over the whole strain range. It is worth mentioning that the HHDP alloy, despite its high yield strength, exhibits exceptional work hardening capability (Figure 2c), which can compensate for the decrease in load‐bearing capacity caused by geometric softening. This unique characteristic endows the HHDP alloy with an unprecedented product of ultimate tensile strength and uniform elongation at an ultrahigh strength level, showcasing a distinct advantage compared to other high‐performance alloys in the literature (Figure 2d).^[^ ^2^, ^15^, ^16^, ^17^, ^18^, ^19^, ^20^, ^21^, ^22^, ^23^, ^24^, ^25^, ^26^, ^27^, ^28^, ^29^, ^30^, ^31^, ^32^, ^33^, ^34^, ^35^, ^36^, ^37^ ^]^ The combination of ultrahigh yield strength and ultimate tensile strength, together with exceptional work hardening, enables the alloy to maintain superior resistance to overload under high‐load service conditions, offering tremendous potential for technological applications.

### 2.3 In Situ Deformation Mechanisms

**Figure 3:**
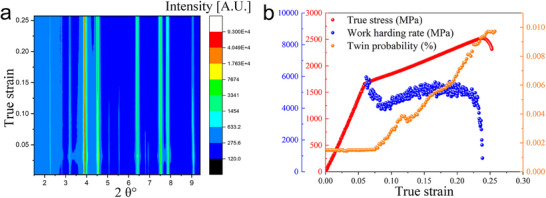
In situ HES‐XRD study of microscopic deformation behavior. a) HES‐XRD pattern during in situ tension of the HHDP alloy. b) Variations of true stress, work hardening rate, and twin probability during in situ synchrotron tensile loading.

To reveal the underlying mechanism responsible for the unusually large work hardening of the ultrastrong HHDP alloy, we used in situ high‐energy synchrotron X‐ray diffraction (in situ HES‐XRD) to characterize the micro‐mechanical behavior and microstructural evolution of the alloy during tensile deformation. **Figure** **3**a displays the evolution of the maximum diffraction intensity as a function of strain during tensile deformation, and Figure 3b shows the true stress–strain curve (red) and the corresponding strain hardening rate curve (blue). The twin probability (*β*) during deformation was calculated based on diffraction peak asymmetry (see Experimental Section), and the results are displayed in Figure 3b (orange) as a function of true strain. It is evident that the probability of twinning starts to increase when the material is deformed by 2% after yielding, indicating that the HHDP alloy activates mechanical twinning at an early stage of plastic deformation. Upon further deformation, the twin probability continues to rise until fracture, implying a dynamic evolution of deformation twinning during the plastic deformation process. It is known that the formation of mechanical twins is highly dependent on the stacking‐fault energy (SFE) and microstructure of materials. Generally, deformation twinning occurs in materials with an SFE of 20–50 mJ m^−2^, whereas dislocation slip occurs in materials with an SFE greater than 50 mJ m^−2^. To probe the physical origin of deformation twinning in the HHDP alloy, we measured the SFE of the FCC matrix using the extended dislocation measurement method. Surprisingly, we found that the SFE of the FCC phase (66 ± 11 mJ m^−2^) is much higher than 50 mJ m^−2^, indicating that the activation of mechanical twinning in the HHDP alloy should be quite difficult. Additionally, the high‐density L1_2_ nanoparticles greatly reduce the spacing of the FCC matrix channels to only a few tens of nanometers, which further increases the critical stress required for mechanical twinning. To quantitatively understand the twinning mechanism, we calculated the critical stress for mechanical twinning in the HHDP alloy. We found that the value is as high as ≈2.6 GPa (see Supporting Information), which is unlikely to be achieved in conventional alloys. Normally, the high SFE and critical twin formation stress would strongly inhibit partial dislocations, making the activation of deformation twinning extremely difficult. To the best of our knowledge, this is the first time that mechanical twinning has been activated in precipitation‐strengthened high‐entropy alloys with such a high SFE at ambient temperature, which is highly unusual but scientifically interesting.

## 3 Discussion

**Figure 4:**
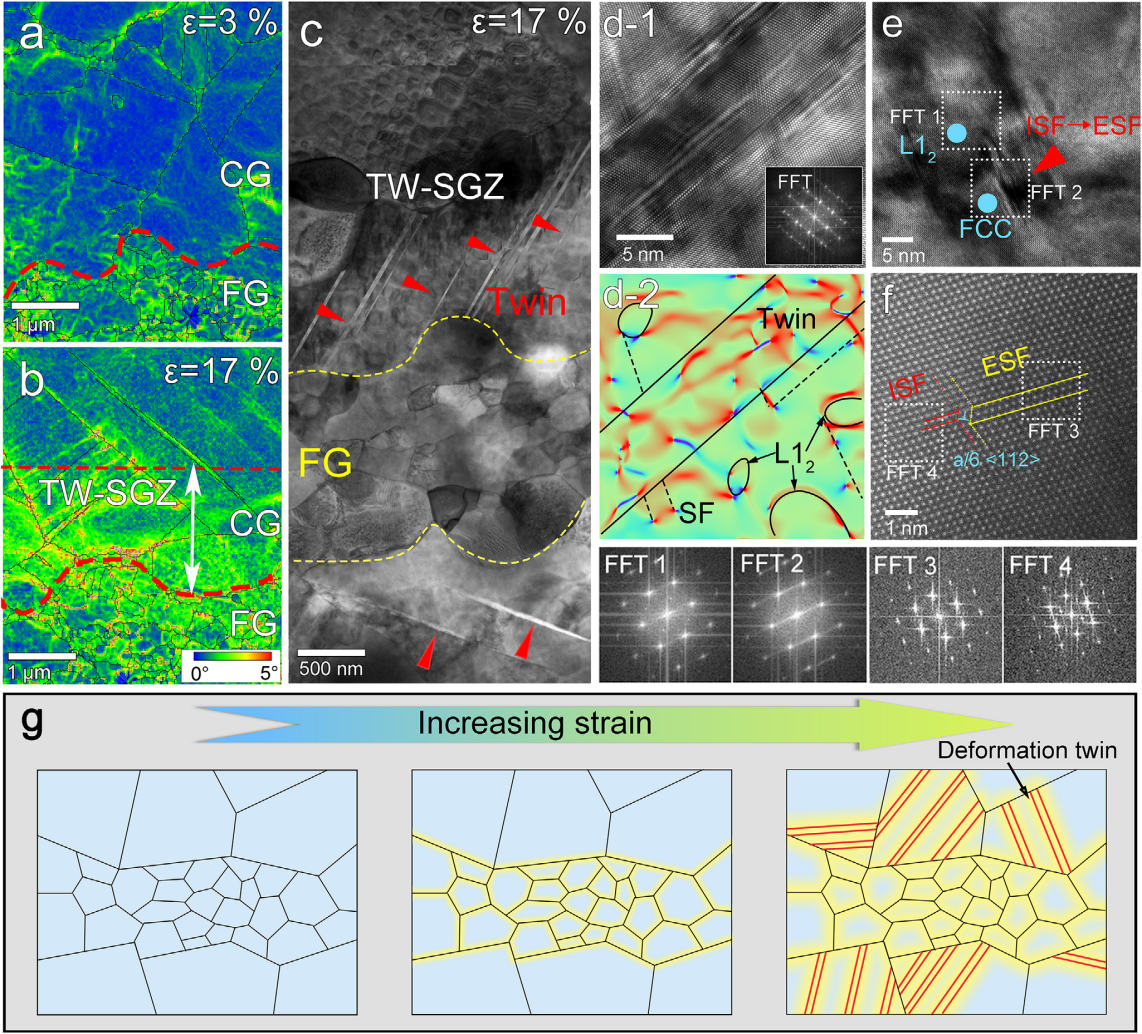
Micro‐deformation mechanisms of the HHDP alloy. a,b) EBSD images of deformed specimens at 3% and 17% tensile strains, respectively. c) TEM image of a deformed specimen at 17% tensile strain, showing the formation of deformation twins in the strain gradient zone (TW‐SGZ) near the interface between the coarse‐grained and ultrafine‐grained regions. d,e) HR‐TEM image and corresponding strain map from geometric phase analysis (GPA) of deformation twins. The corresponding FFT pattern, showing twin reflections, is displayed in the inset. f) HR‐TEM image showing the transition from ISFs to ESFs. g) HR‐STEM image illustrating that the interaction of *a*/6<112> type glissile dislocations, through successive passage on the (111) planes, triggers the formation of ESFs. h) Schematic diagrams illustrating microstructural evolution with increasing strain.

As described above, we achieve an unprecedent combination of ultrahigh yield strength, large ductility, and exceptional work hardening in a multicomponent alloy through hierarchically heterogeneous architecting, which induces deformation twinning as an additional mechanism for enhancing plastic deformation stability. To probe the structural origins of the exceptional work hardening in the HHDP alloy, we used EBSD and TEM to study the dynamic evolution of deformation substructures under different strains, and the results are displayed in **Figure** **4**. At the early stage of deformation (3% strain), the HHDP alloy exhibits heterogeneous deformation between the coarse‐grained and ultrafine‐grained regions (Figure 4a), with average KAM values of 0.22° and 0.77°, respectively. This substantial difference indicates that plastic strain is preferentially accommodated in the ultrafine‐grained regions, reflecting a pronounced strain partitioning effect. As a result, a strain gradient zone forms near the interface between the two regions to accommodate the mismatch in deformation. As deformation progresses, both the ultrafine‐grained and coarse‐grained regions undergo work hardening, as evidenced by the increase in average KAM values to 0.97° for the coarse‐grained region and 2.31° for the ultrafine‐grained region at 17% strain. Despite the overall hardening, the ultrafine‐grained region remains significantly harder than the coarse‐grained region. This persistent disparity underscores the ongoing strain partitioning and the continued development of the strain gradient zone (Figure 4b). Moreover, geometrically necessary dislocation (GND) density analysis reveals a smooth transition from the ultrafine‐grained region to the coarse‐grained region across the interface (Figure S6), clearly illustrating the characteristics of the strain gradient zone. Notably, a high density of nanotwins was observed predominantly in the strain‐gradient zones, with a twin area fraction of ≈23% and an average twin thickness of 9 ± 2 nm. A representative TEM image in Figure 4c shows that multiple parallel nanotwins are formed in the strain‐gradient zone. HR‐TEM imaging (Figure 4d) clearly reveals the atomic structure of a representative deformation twin, confirming the in situ HES‐XRD results. Using HR‐STEM (Figure 4f), we observed that the deformation twins are formed through a transition from intrinsic SFs (ISFs) to extrinsic SFs (ESFs), resulting from the motion of a partial dislocation of the 1/6<112> type on the continuous (111) plane. We schematically present the formation of deformation twins in the strain‐gradient region in Figure 4h, which is closely related to the unique hierarchical heterostructure of the alloy. In contrast, only SFs and dislocations are detected in the reference alloys with a homogeneous grain structure, without any observation of deformation twins (Figure S7), further confirming that the hierarchical heterostructure is crucial for the deformation twinning of the HHDP alloy.

On the basis of the above observations, we now discuss the underlying mechanisms responsible for the unusual formation of mechanical twinning in the HHDP alloy with an ultrahigh yield strength of more than 1.5 GPa. First, the HHDP alloy has heterostructures in terms of both grain and precipitate sizes, which can provide ultrahigh stresses in localized regions through hetero‐deformation. Generally, deformation twinning can be triggered when a threshold value of the critical twinning stress is exceeded. In this alloy, the high SFE and nanoprecipitates significantly elevate the critical twinning stress to 2.6 GPa. Apparently, the applied stress of the HHDP alloy during the tensile process can not reach this value. However, it is worth noting that the ultrafine‐grained regions have a much higher strength than the coarse‐grained regions due to the Hall‐Petch effect. As a result, GNDs are blocked by and pile up against the interface between the two regions, which results in the generation of localized ultrahigh stresses in the strain‐gradient zone. Therefore, the hetero‐deformation‐induced localized ultrahigh stresses in the strain‐gradient zone can provide the necessary stress for the activation of mechanical twinning. Notably, the deformation twins are formed predominantly within these strain gradient zones. This spatial confinement demonstrates that hetero‐deformation‐induced strain gradients and the resulting localized ultrahigh stresses play a crucial role in the formation of deformation twins. Second, the formation of deformation twinning in the HHDP alloy is associated with the transition of SFs to twins. Specifically, the SFE controls the nucleation of ISF through *a*/6<112> type glissile Shockley partial dislocations in the FCC matrix and super‐partials of *a*/3<112> type in the L1_2_ precipitates. The interaction of *a*/6<112> type glissile dislocations via successive passage on the (111) planes triggers the formation of ESFs, which subsequently thicken into nanotwins (Figure 4f,g). In other words, the plastic deformation of the HHDP alloy is accommodated primarily through the transformation of two‐layer ISFs into three‐layer ESFs, leading to the formation of deformation twins. Consequently, the combination of the hetero‐deformation‐induced ultrahigh stress in the strain‐gradient zone and the transition of ISFs to ESFs leads to the unusual formation of deformation twins in the HHDP alloy with ultrahigh yield strength.

We now explain in more detail the underlying factors contributing to the exceptional work hardening and high ductility of the ultrastrong HHDP alloy. First, deformation‐induced twins are formed in the strain‐gradient zone, which reduces the mean free path of dislocations, resulting in a strengthening effect commonly referred to as the “dynamic Hall‐Petch” phenomenon. As illustrated in Figure 4d, mechanical twins serve as formidable impediments to dislocation slip and consequently enhance the work hardening of the alloy. The contribution of mechanical twinning to work hardening is explicitly demonstrated in Figure 2b. Second, the twin‐dominated strain‐gradient zone exhibits excellent dislocation storage capability, which effectively channels high stresses from the ultrafine‐grained regions into the strain‐gradient zone. As such, we obtained a high density of GNDs and unusually high strength in the strain‐gradient zone, thereby achieving simultaneous enhancement of both strength and ductility. Third, since the coarse‐grained and ultrafine‐grained regions differ in strength, hetero‐deformation produces long‐range internal stress, i.e., back stress, in the coarse‐grained regions. The back stress is directional and offsets some applied shear stress, which makes the coarse‐grained regions appear stronger to sustain higher applied stresses, leading to the hetero‐deformation‐induced (HDI) work hardening. Through experiments, we examined the contribution of HDI hardening to the true stress of the alloy (Figure S5). We found that the contribution of HDI stress to work hardening is ≈487 MPa at 17% strain. Therefore, HDI work hardening is also an important factor contributing to the exceptional work hardening of the HHDP alloy. Furthermore, deformation of the non‐strain‐gradient zone in the coarse‐grained regions is dominated by SFs. The intersection of SFs along two (111) slip planes results in the formation of nanoscale SF networks (Figure S11a), which divide the matrix into nanoscale domains. HR‐TEM reveals the formation of a high density of sessile Lomer‐Cottrell (L‐C) locks at the intersections of SFs (Figure S11b), which results from the reaction of two parts of dissociated dislocations. Both the nanoscale SF networks and L‐C locks are effective barriers to dislocation motion, thereby contributing to the progressive and steady work hardening of the HHDP alloy.

Therefore, it is evident that the activation of unusual deformation twinning contributes significantly to the superior work hardening capability and plastic deformation stability of the ultrahigh‐strength HHDP alloy. Additionally, with the formation of mechanical twins in the strain‐gradient zone, the stress concentration in the ultrafine‐grained regions is relieved, and dislocation storage capacity in the coarse‐grained regions is enhanced (Figure 4h). In association with hetero‐deformation, the HHDP alloy also exhibits HDI work hardening. Furthermore, the formation of nanoscale SF networks in the coarse‐grained regions acts as a strong barrier to dislocation motion, which further enhances the work hardening of the alloy. As a result, our HHDP alloy exhibits excellent work hardening and ductility at an ultrahigh yield strength level of more than 1.5 GPa.

In summary, we have architected the HHDP alloy with a unique hierarchical heterostructure, which activates deformation twinning as an additional mechanism for enhancing the work hardening and ductility of this ultrahigh‐strength alloy. This unique structural design endows our alloy with an ultrahigh yield strength (1.5 GPa) and superior work hardening capability (≈6 GPa work hardening rate), imparting a tensile strength of ≈2.2 GPa and a uniform ductility of ≈20%. Deformation twins are extensively activated in the strain‐gradient zone, which facilitates the dynamic Hall‐Petch effect, contributing substantially to the work hardening and plastic deformation stability of the alloy. Additionally, the strain‐gradient zone demonstrates excellent dislocation storage capacity, effectively transferring stress concentrations from the ultrafine‐grained regions to the coarse‐grained regions. Our newly developed HHDP alloy, with its unprecedented combination of ultrahigh yield strength, ductility, and work hardening capability, should have tremendous potential for technological applications, such as aerospace, automotive, and energy industries. Our strategy of hierarchical heterostructure architecting can be applied to many other materials, such as superalloys, titanium alloys, and aluminum alloys, to achieve synergistic mechanical properties for structural applications.

## 4 Experimental Section

### Sample Preparation

An alloy ingot of Ni_38_Co_25_Fe_13_Cr_10_Al_7_Ti_7_ (at.%) was prepared by arc melting in an argon atmosphere and subjected to multiple remelting cycles to ensure chemical homogeneity. The raw materials used comprised commercially pure metals with a purity exceeding 99.95 wt.%. Using copper mold casting technology in a high‐purity argon atmosphere, a plate sample with dimensions of 8 mm × 12 mm × 50 mm was produced. The sample underwent heat treatment at 1150 °C for 2 h, followed by rapid quenching in water. After cold rolling, the sample was annealed at 1080 °C for 90 s to obtain heterogeneous grain structures. Subsequently, the sample was subjected to aging treatments at 780 °C for 16 h, followed by an additional aging step at 650 °C for 24 h (referred to as the HHDP alloy). For comparison, two samples with the same composition as the HHDP alloy but different treatment processes were prepared; one was annealed at 1100 °C for 30 min following cold rolling, resulting in a fully recrystallized equal‐axis crystal structure (referred to as the “as‐solutionized sample”), and the other, derived from the as‐solutionized sample, was further aged at 780 °C for 16 h and subsequently at 650 °C for 24 h (referred to as the “solutionized and aged sample”).

### Tests and Characterization

Uniaxial tensile testing was performed using flat dog‐bone specimens (0.8 mm × 2.5 mm × 16 mm) on an electronic universal testing machine (Instron 5582) to assess the tensile properties. The tests were performed at a constant strain rate of 10^−3^ s^−1^ with a data acquisition rate of 10 Hz, and the actual strain was measured using a contact extensometer. The microstructure of the specimens was characterized using a scanning electron microscope (SEM; XL30‐FEG, Holland) with an EBSD detector operating at a working voltage of 20 kV. EBSD samples were subjected to electrolytic etching in a solution consisting of 10% perchloric acid and 90% ethanol at 20 V and −20 °C for 60 s. For a more detailed examination of the microstructure and composition, TEM (Talos F200X) equipped with an EDX detector was employed. Atomic‐resolution HAADF‐STEM imaging was executed using a Cs probe‐corrected and monochromated field‐emission gun (FEG) Spectra 300 STEM, operated at an acceleration voltage of 300 kV. TEM samples were prepared using a twin‐jet electropolishing machine with a 10% perchloric acid ethanol solution at 25 V and −30 °C, resulting in a thickness of ≈50 nm in the electron‐transparent region.

### In Situ Synchrotron XRD Tensile Testing

In situ tensile tests were conducted using dog‐bone specimens on a universal testing machine with a maximum load capacity of 20 kN. A high‐energy beam of 87 keV (corresponding to a wavelength of 0.14235 Å) was employed, enabling operation in transmission mode for the acquisition of bulk microstructure information. The beam dimensions were set to 700 × 700 µm^2^. The material was loaded to predefined strains, enabling the analysis of both elastic and plastic behavior of the alloy. A PerkinElmer 2D detector with a pixel size of 200 × 200 µm^2^ was utilized to capture 2D raw Debye‐Scherrer rings at various stress/strain levels. Each loading step had an exposure time of 0.2 s, and 10 images were captured, including dark images for background subtraction. Dark images were subtracted from each image to significantly minimize residuals from prior exposures. Prior to in situ testing, LaB_6_ was employed to determine the peak broadening associated with the beamline and optics, as well as to establish the sample‐to‐detector distance (calculated as 1226 mm).

## Conflict of Interest

The authors declare no conflict of interest.

## Data Availability

The data that support the findings of this study are available from the corresponding author upon reasonable request.
